# Complications of the suburethral sling in the form of Mini Vaginal Tape (MVT): A case report

**DOI:** 10.1016/j.ijscr.2022.106946

**Published:** 2022-03-16

**Authors:** Md. Mominul Islam, Md. Saiful Islam, A.K.M. Khurshidul Alam, Mohammad Monsur Hallaz

**Affiliations:** a Indoor medical officer (KGH), Ex-Resident, Department of Urology, Bangabandhu Sheikh Mujib Medical University, Bangladesh; b Female androurologist, Department of Urology, Bangabandhu Sheikh Mujib Medical University, Bangladesh; c Department of Urology, Bangabandhu Sheikh Mujib Medical University, Bangladesh; d Department of Urology, Ibn Sina Medical College Hospital, Bangladesh

**Keywords:** Case report, Mid urethral sling (MUS), Mini vaginal tape (MVT), Sub urethral sling, Peri-urethral stone, Urethro-vaginal fistula (UVF), Tension-free vaginal tape (TVT), Transobturator tape (TOT)

## Abstract

Synthetic sub-urethral sling has become the most widely used technique for the surgical management of stress urinary incontinence. Despite a higher success rate, complications like migration, encrustation, and stone formation have been reported by a mid-urethral sling (MUS). Among mid-urethral sling procedures, mini vaginal tape (MVT) is very popular. As periurethral stone formation and urethrovaginal fistula are very uncommon after MVT, case report on this issue is sparse. The current case report features a 55-year female presented with lower abdominal pain and continuous urinary incontinence, 10 years after the MVT. She was diagnosed as a case of periurethral stone with urinary incontinence due to urethro-vaginal fistula. Our surgical team successfully removed the stone formed by the encrustation of the displaced tape and repaired the fistula. Following the MVT, a high degree of suspicion and long-term follow-up is mandatory for the diagnosis and management of these rare complications.

## Highlights

• Mini vaginal tape (MVT), a form of synthetic mid-urethral sling is popular for treating stress urinary incontinence.
• There are limited reports on periurethral stone formation and urethrovaginal fistula by MVT as it is rare.
• This study presents a case of periurethral stone in the MVT with urinary incontinence due to urethrovaginal fistula.
• Although rare, a high degree of suspicion is mandatory for the diagnosis and management of these rare complications.

## 1 Introduction and importance

Sub urethral sling was first described in 1995 as a surgical treatment of stress urinary incontinence. This sling is placed in the mid urethra as the pubourethral ligament is functionally inserted in this region. [1]. Surgeons prefer to administer mid-urethral sling in the form of mini vaginal tape (MVT) and it is considered as the gold standard procedure [2]. But it may lead to complications- like per-operative bladder or bowel perforation, urethral erosion, urethro-vaginal fistula formation [3]. There are few case reports regarding accidental bladder perforation and intravesical stone formation [4], [5], [6]. The current study presents a case of encrustation and urethro-vaginal fistula formation after 10 years of mini vaginal tape (MVT) procedure. It was successfully managed by a transvaginal surgical approach.

## 2 Case presentation

A 55 years old female presented with continuous pervaginal urinary leakage with lower abdominal pain. She was normotensive, non-diabetic, had a non significant drug history. This lady was a nonsmoker and mentally sound but anxious about her recent urinary complaints. She had a history of traumatic forceps delivery at her first pregnancy due to stillbirth, 25 years back. Following the procedure, she developed stress incontinence but did not take any surgical management for it. Subsequently, she underwent three lower uterine caesarian sections (LUCS) and total abdominal hysterectomy with bilateral salpingo-oophorectomy during her last LUCS, 18 years back. For stress urinary incontinence (SUI), sub urethral sling was inserted in a tertiary hospital in the form of mini vaginal tape (MVT), 10 years back. Following this procedure, she was dry for 5 years. Then she experienced continuous urinary leakage through the vagina, which had been deteriorating over the previous three months. The patient complained of hard vaginal mass and pain in her lower abdomen for the last 2 months. Initially, vaginoscopy was performed under local anesthesia. External urethral meatus was found normal, but a fixed stony hard mass was noted in the anterior vaginal wall with a fistula tract. X-ray KUB of this patient-reported an irregular 3 × 5 cm radio-opaque shadow in the bladder neck area. An ultrasound scan (USG) documented the stone and the urethrovaginal fistula. Subsequently, cystoscopy was performed with regional anesthesia (subarachnoid block) for confirmation of the diagnosis and further management plan.

**Fig. 1:**
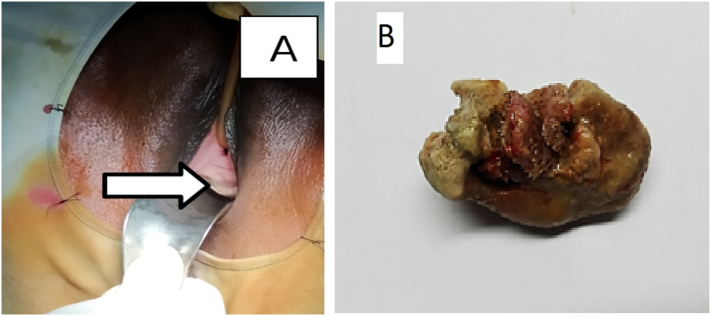
A: Stone marked by an arrow in peri-urethral region. B: Postoperative view of removed MVT (Minivaginal tape) stone.

Cystoscopy found a large stone, eroding in the urethra just distal to bladder neck [Fig. 1(A)] and a large urethro-vaginal fistula tract. The bladder neck was wide open and eroded, both ureteric orifices were pushed laterally, rest of the bladder wall was normal. After securing both ureters by 6 Fr. double J(DJ) ureteral stent, stone [Fig. 1(B)] was removed at lithotomy position by sharp and blunt dissection through the vaginal route. Then the patient's position was changed, and the urethro-vaginal fistula was repaired in 3 layers at the Jack-knife position keeping a 16fr catheter in situ. Postoperative progress was uneventful. Follow up schedule was 3 weekly up to 6 months. This case report was conducted according to SCARE 2020 guidelines [7]. Written informed consent was obtained from the patient for publication of this case report and accompanying images.

## 3 Clinical discussion

Synthetic mid-urethral sling is a widely used surgical treatment of stress urinary incontinence. This is a less invasive procedure and has a high success rate [2]. However, it is not free of complications [4], [5], [8].

Tension-free vaginal tape (TVT), transobturator tape (TOT), minivaginal tape (MVT) are types of mid-urethral sling. Mini vaginal tape (MVT) placement procedure is very simple. A 1.5 cm × 1 cm tape is placed in suburethral space by anchoring it with the inferior pubic rami by a 3/0 absorbable suture (polyglactin). In this procedure smaller needles and tape are used thus the chance of neurovascular bundle injury is extremely low.

Tape-related complications are increasing and at present, it is approximately 6% [3]. Limited studies reported accidental vesical perforation by mid-urethral sling and stone formation in the intravesical part but stone formation in the periurethral space is rare. [4], [5], [6]. The current case report is regarding stone formation in peri-urethral space with the formation of a urethro-vaginal fistula by the mini vaginal tape.

All mid-urethral slings use self-anchoring tape, but mini vaginal tape (MVT) is the exception. As it is fixed with pubic rami, repeated per vaginal intervention can displace the sling and result in erosion of the urethra. This made the sling vulnerable to exposure with urine. The synthetic mesh exposed to urine works as a nidus for encrustation, which gradually increases in size and forms stone [6], [9], [10]. In the current case, the gradual increase of stone size and erosion of mucosa resulted in urethrovaginal fistula. This fistula disrupted the sphincteric function and was close to the bladder neck resulting in incontinence of this patient. The female andro-urology surgical team of Bangabandhu Sheikh Mujib Medical University (BSMMU) removed the encrusted tape and successfully repaired the fistula. She was discharged from the hospital on the 10th postoperative day and the urethral catheter was removed on the 21st postoperative day. The patient was in regular follow up upto 6 months and found dry with no urinary complaints.

## 4 Conclusion

Mini vaginal tape (MVT) is a very simple, minimally invasive procedure, but it may end up with complications. In this case, the patient presented with a urethrovaginal fistula and continuous urinary incontinence with the formation of stone in the tape. So, postoperative long-term follow-up with high suspicion is cardinal for diagnosis and management of these rare complications.

## Abbreviation

LUCS: lower uterine caesarian section
MUS: mid urethral sling
MVT: mini vaginal tape
SUI: stress urinary incontinence
TVT: tension-free vaginal tape

## Provenance and peer review

Not commissioned, externally peer-reviewed.

## Source of funding

No funding was allocated for this study.

## Ethical approval

The study is exempted from the ethical approval of the institute. This case report does not involve any sample from the human body parts like blood, tissue, or any organ of the body.

## Consent

Written informed consent was obtained from the patient for publication of this case report and accompanying images. A copy of the written consent is available for review by the Editor-in-Chief of this journal on request.

## Author contribution

Md. Mominul Islam- study concept and design, data collection, writing the paper, operative team member.

Md. Saiful Islam- study design, data analysis, data interpretation, writing the paper, operative team leader.

AKM Khurshidul Alam- study design, data analysis, and interpretation, writing the paper.

Mohammad Monsur Hallaz-data analysis and interpretation, writing the paper.

## Research registration

Obtained from Researchregistry.com, Research Registry's unique identifying number (UIN) of this study is 7730.

## Guarantor

Guarantor is Md. Mominul Islam.

I, Md Mominul Islam, accept full responsibility for the work and/or the conduct of the study article, full access to data, and controlling decisions about publishing the paper.

## Declaration of competing interest

No conflict of interest found.
